# Biochemical progression free and overall survival among Black men with stage IV prostate cancer in South Africa: Results from a prospective cohort study

**DOI:** 10.1002/cam4.6739

**Published:** 2023-12-29

**Authors:** Yoanna S. Pumpalova, Adarsh Ramakrishnan, Michael May, Audrey Pentz, Shauli Minkowitz, Sean Doherty, Elvira Singh, Wenlong Carl Chen, Timothy R. Rebbeck, Alfred I. Neugut, Maureen Joffe

**Affiliations:** ^1^ Department of Medicine, Vagelos College of Physicians and Surgeons Columbia University New York City New York USA; ^2^ Department of Epidemiology, Mailman School of Public Health Columbia University New York City New York USA; ^3^ Strengthening Oncology Services Research Unit, Faculty of Health Sciences University of the Witwatersrand Johannesburg South Africa; ^4^ Division of Urology, Department of Surgery, Faculty of Health Sciences University of the Witwatersrand Johannesburg South Africa; ^5^ National Cancer Registry National Health Laboratory Service Johannesburg South Africa; ^6^ Sydney Brenner Institute for Molecular Bioscience Faculty of Health Sciences, University of the Witwatersrand Johannesburg South Africa; ^7^ Dana Farber Cancer Institute Boston Massachusetts USA; ^8^ Harvard T.H. Chan School of Public Health Boston Massachusetts USA; ^9^ Herbert Irving Comprehensive Cancer Center, Vagelos College of Physicians and Surgeons Columbia University New York City New York USA

**Keywords:** cancer management, hormone therapy, metastasis, prostate cancer

## Abstract

**Background:**

Men of African descent are disproportionately affected by prostate cancer (PCa), and many have metastatic disease at presentation. In South Africa (SA), androgen deprivation therapy (ADT) is the first‐line treatment for stage IV PCa.

**Objective:**

To identify predictors of overall survival (OS) in Black South African men with stage IV PCa treated with ADT.

**Design, Setting, and Participants:**

Men diagnosed with prostate cancer (3/22/2016–10/30/2020) at Chris Hani Baragwanath Academic Hospital in Soweto, Johannesburg, were recruited for the Men of African Descent with Cancer of the Prostate study. We included men with newly diagnosed stage IV PCa treated with ADT who had a prostate‐specific antigen (PSA) level drawn prior to initiation of ADT and had ≥1 PSA drawn ≥12 weeks after ADT start.

**Outcomes Measures and Statistical Analysis:**

We used Kaplan–Meier statistics to estimate OS and Cox regression models to identify predictors of OS.

**Results and Limitations:**

Of the 1097 men diagnosed with prostate cancer, we included 153 men with stage IV PCa who received ADT and met PSA requirements. The median age was 68.0 years (interquartile range 64–73 years). Median OS from time of ADT initiation was 3.39 years (95% confidence interval (CI): 3.14%–noncalculable), while biochemical progression‐free survival was 2.36 years (95% CI: 2.03%–3.73%). Biochemical progression (HR 3.52, 95% CI: 1.85%–6.70%), PSA nadir level >4 ng/mL (HR 3.77, 95% CI: 1.86%–7.62%), alkaline phosphatase level at diagnosis >150 IU/dL (HR 3.09, 95% CI: 1.64%–5.83%), and hemoglobin at diagnosis <13.5 g/dL (HR 2.90, 95% CI: 1.28%–6.56%) were associated with worse OS.

**Conclusions:**

In this study, we identified factors associated with poor OS among Black South African men with stage IV PCa treated with ADT. These factors may be useful in identifying patients for upfront treatment escalation, including the use of docetaxel chemotherapy or escalation of therapy at the time of biochemical progression.

**Patient Summary:**

In this study, we found that high alkaline phosphatase level, anemia at diagnosis, and high PSA nadir after initiation of androgen deprivation therapy are associated with worse overall survival among Black South African men treated with androgen deprivation therapy for metastatic prostate cancer.

## 1 BACKGROUND

Prostate cancer (PCa) is the second most common cancer and the fifth most common cause of cancer death in men worldwide, and it was responsible for 6.8% of all cancer‐related deaths in men in 2020.^1^ In addition to increasing age and a family history of PCa, African ancestry is one of the few known risk factors for PCa.^2^, ^3^, ^4^ Studies from Sub‐Saharan Africa (SSA) have shown a rising PCa incidence and a high percentage of men presenting with stage IV PCa. Reports from Burkina Faso, Togo, Nigeria, Kenya, Senegal, and the Ivory Coast show that 67%–99% of patients presented with advanced or metastatic PCa.^5^

The age‐standardized risk (ASR) for PCa in SA was 52.73 per 100,000 men in 2019.^6^ White men have the highest risk, with an estimated ASR of 77 per 100,000 in 2017, compared to 45 per 100,000 in Black men.^6^ This may reflect a true difference in incidence or may be due to differential access to diagnosis and screening in the Black population, who experience a myriad of barriers to healthcare access and frequently receive care in the low‐resourced public healthcare sector in SA.

In SA, first line of treatment for Stage IV PCa is androgen‐deprivation therapy (ADT) with luteinizing hormone‐releasing hormone (LHRH) agonists and/or oral non‐steroidal first‐generation antiandrogens, with or without bilateral orchiectomy.^7^ In the public healthcare sector, second‐generation anti‐androgen agents are not available, and chemotherapy is grossly underused. Studies from high‐income countries show that among men who receive first‐line monotherapy with medical ADT and/or orchiectomy for Stage IV PCa, many will develop castrate resistance and die from PCa.^8^, ^9^ The rate of progression to castrate‐resistant PCa and death among Black African men with Stage IV PCa treated with ADT is poorly understood. Understanding the demographic and clinical factors associated with progression to castrate resistance and/or death in this population will allow clinicians to select patients who may benefit from more aggressive upfront therapy.

In this study, we estimated the overall survival (OS) of Black African men diagnosed with stage IV PCa in SA and determine the demographic and clinical factors associated with OS. Our secondary aim was to determine the biochemical progression‐free survival (bPFS) and to evaluate if biochemical progression is correlated with OS.

## 2 METHODS

### 2.1 Data source

The MADCaP Consortium is a case–control study of men of African ancestry with PCa and age‐matched controls.^10^, ^11^ This analysis is restricted to men enrolled in MADCaP at the Chris Hani Baragwanath Academic Hospital (CHBAH) in Soweto, Johannesburg, SA.

### 2.2 Patient population

We included men ≥30 years of age of African descent enrolled in MADCaP and receiving care at the CHBAH site in Soweto, Johannesburg, between March 22nd, 2016 and October 30th, 2020. Soweto is a township in Johannesburg; its population is 98.5% Black and has one of the highest rates of poverty in the region.^12^ Included patients had stage IV PCa with no prior cancer diagnosis and had lived in the greater Soweto area for ≥10 years. Stage IV PCa was defined as invasive adenocarcinoma of the prostate on prostate biopsy and evidence of distant metastasis on nuclear bone scan and/or other imaging within 6 months of diagnosis, or invasive prostate adenocarcinoma on biopsy of a distant metastatic site. Men with incomplete metastatic workups were excluded. We included only patients who underwent bilateral orchiectomy and/or received ≥1 dose of medical ADT, had a prostate‐specific antigen (PSA) level drawn prior to or on the day of ADT start, and had ≥1 PSA level drawn ≥12 weeks after ADT start.

### 2.3 Data collection

We collected demographic, clinical, and outcomes data on each subject, including age at diagnosis, self‐reported race, marital status, body mass index (BMI), waist circumference, number of years of education, income, self‐reported history of cardiovascular disease (diabetes mellitus, hypertension, hyperlipidemia, ischemic heart disease, and stroke), HIV status, smoking history, and family history of PCa. PCa symptoms and Patients' International Prostate Symptom Score (IPSS) at diagnosis were recorded.^13^ Gleason group, presence of lymphovascular and perineural invasion (LVI/PNI), and tumor volume (as a percent of total prostate volume) were obtained from pathology reports. Clinical T‐stage was determined by digital rectal exam.

Prostate‐specific antigen, alkaline phosphatase (ALP), calcium, and hemoglobin (Hgb) levels were obtained at enrollment. Management protocol at CHBAH included PSA level at time of diagnosis and every 6–12 months. Treatment protocol consisted of ADT with a LHRH agonist (goserelin or buserelin) every 3 months. Oral non‐steroidal antiandrogens (flutamide or bicalutamide) were prescribed to symptomatic patients. Bilateral orchiectomy was considered in patients who were unable or unwilling to come in for injections.

Treatment and survival outcomes data were collected through June 30, 2021. Patients were contacted every 3–6 months to determine vital status. If the patient or identified person of contact were unable to be reached for two consecutive follow‐up calls, we searched publicly available administrative data to determine vital status for that patient. If no additional information about vital status could be obtained, the patient was censored at last known follow‐up date.

### 2.4 Survival and biochemical progression

Overall survival was defined as time from bilateral orchiectomy or ADT initiation (ADT start) to death from any cause (event) or last date when patient was confirmed to be alive (censored). Biochemical progression‐free survival was defined as time from ADT start to biochemical progression (event) or date when last PSA level was drawn (censored). For patients who had an initial decline in PSA level after ADT relative to the PSA drawn prior to or on day 1 of ADT, post‐treatment PSA nadir was defined as the lowest post‐treatment PSA prior to a subsequent increase in PSA, or last drawn PSA if there was no documented increase.

Biochemical progression event was defined as any of:

1. First PSA value drawn ≥12 weeks after ADT start that was ≥2 ng/mL and ≥ 25% compared to posttreatment PSA nadir,
2. First PSA value drawn ≥12 weeks after ADT start that was ≥2 ng/mL and ≥ 25% compared to PSA at ADT start, if there was no nadir,
3. First PSA value drawn ≥12 weeks after ADT start that was ≥5 ng/mL and ≥ 50% compared to PSA level prior to ADT start, if there was no nadir and and PSA was not drawn at ADT start.

Biochemical progression event definitions were adapted from the Prostate Cancer Working Group 3 recommendations.^14^, ^15^ Key differences include: (1) omitting repeat PSA testing 3 weeks after initial rise to confirm a rising PSA trend and (2) including definition (3) of biochemical progression. These modifications were necessary because the South African public healthcare system does not subsidize more than one PSA level every 3 months.

### 2.5 Statistical analysis

We used descriptive statistics to report baseline demographic and clinical characteristics and Kaplan–Meier statistics to estimate OS.

We tested all variables for association with OS using univariate Cox proportional hazards models and included all variables with a *p*‐value < 0.1 in a multivariate Cox regression model (Table 2). We removed calcium level at diagnosis due to low sample size in the elevated category (*n* = 4). PSA progression was treated as a time‐dependent covariate in all regression models.^16^

All statistical analyses were done using the R version 4.0.5 and SAS version 9.4.

## 3 RESULTS

### 3.1 Baseline characteristics

During the study period, 1097 men with PCa enrolled in MADCaP at CHBAH. We excluded 227 patients with incompletely staged PCa. Out of the remaining 878 patients, we further excluded 678 with localized PCa and included 200 men with stage IV PCa (22.8%). Of these, 8 men never initiated ADT, and 39 men had insufficient PSA measurements to calculate biochemical progression‐free survival. We included 153 men who met the ADT administration and PSA measurement requirements for our study. The median age was 68 years (IQR 64–73 years). All 153 men (100%) identified as Black, 120 (78.4%) had a relatively low income of <1850 ZAR per month (equivalent to <$132), 94 (61.4%) reported a history of ≥1 cardiovascular comorbidity, 11 (7.2%) were HIV positive, and 111 (72.5%) were current or former smokers (Table 1).

**TABLE 1:** Baseline and treatment characteristics of men with stage IV prostate cancer who received ADT and were enrolled in the MADCaP study at the Chris Hani Baragwanath Academic Hospital in Johannesburg, South Africa (2016–2020).

	Overall (*N* = 153)
Age category	
<65 years	41 (26.8%)
>=65 years	112 (73.2%)
Marital status	
Divorced	15 (9.8%)
Married	96 (62.7%)
Single	10 (6.5%)
Widowed	32 (20.9%)
Education level	
>12 years of schooling	4 (2.6%)
0‐4 years of schooling	24 (15.7%)
5‐12 years of schooling	114 (74.5%)
No Formal education	11 (7.2%)
Income	
>10,000 per month	1 (0.7%)
1851–10,000 per month	20 (13.1%)
Chose not to answer	12 (7.8%)
Up to 1850 per month	120 (78.4%)
BMI	
BMI 18.5–24.9	65 (42.5%)
BMI < 18.5	9 (5.9%)
BMI 25.0–29.9	44 (28.8%)
BMI > 30.0	35 (22.9%)
Number of cardiovascular comorbidities^a^	
0	59 (38.6%)
1	59 (38.6%)
2	28 (18.3%)
3	7 (4.6%)
HIV status	
No	142 (92.8%)
Yes	11 (7.2%)
Smoking status	
Current smoker	39 (25.5%)
Former smoker	72 (47.1%)
Never smoker	42 (27.5%)
Family history	
N‐Miss	78
Negative	66 (88.0%)
Positive	9 (12.0%)
Intentional weight loss	
N‐Miss	2
No	124 (82.1%)
Yes	27 (17.9%)
Prostatitis at diagnosis	
No	10 (6.5%)
Yes	143 (93.5%)
IPSS	
Median	9.000
Q1, Q3	5.000, 20.000
IPSS category	
Mild symptoms	67 (43.8%)
Moderate symptoms	44 (28.8%)
Severe symptoms	42 (27.5%)
Lower urinary tract symptoms at diagnosis	
No	38 (24.8%)
Yes	115 (75.2%)
PSA at diagnosis	
Median	71.530
Q1, Q3	20.720, 432.600
PSA at diagnosis, categorized	
<100 ng/mL	84 (54.9%)
>/=100 ng/mL	69 (45.1%)
Tumor volume (%)	
N‐Miss	1
<50%	57 (37.5%)
>=50%	95 (62.5%)
Gleason GROUP	
Median	5.000
Q1, Q3	3.000, 5.000
LVI at diagnosis	
N‐Miss	15
No	97 (70.3%)
Yes	41 (29.7%)
PNI at diagnosis	
No	37 (24.2%)
Yes	116 (75.8%)
Clinical T‐stage	
T1	29 (19.0%)
T2	93 (60.8%)
T3	28 (18.3%)
T4	3 (2.0%)
Alkaline phosphatase at diagnosis	
N‐Miss	1
</=150 IU/L	102 (67.1%)
>150 IU/L	50 (32.9%)
Hemoglobin at diagnosis	
N‐Miss	2
>=13.5 g/dL	63 (41.7%)
<13.5 g/dL	88 (58.3%)
Calcium at diagnosis	
N‐Miss	1
</= 2.7 mmol/L	150 (98.7%)
>2.7 mmol/L	2 (1.3%)
Bone only metastatic disease	
N‐Miss	14
No	6 (4.3%)
Yes	133 (95.7%)
Underwent bilateral orchiectomy	
No	142 (92.8%)
Yes	11 (7.2%)
PSA nadir	
Median	2.820
Q1, Q3	0.550, 17.930

Abbreviations: HIV, human immunodeficiency virus; IPSS, international prostate symptom score; LHRH, luteinizing hormone releasing hormone; PSA, prostate‐specific antigen.

^a^ Included history of stroke, diabetes mellitus, ischemic heart disease, hypertension, and hyperlipidemia.

Most patients had a history of prostatitis (93.5%) and/or presence of lower urinary tract symptoms (LUTS) at diagnosis (75.2%). At diagnosis, 69 patients (45.1%) had PSA levels ≥100 ng/mL, with a median PSA of 71.5 ng/mL (IQR 20.72–432.60) (Table 1). Of the 153 patients in our cohort, 57.5% had Gleason Group 5 PCa, 29.7% had LVI, and 75.8% had PNI on diagnostic biopsy.

### 3.2 Treatment

All 153 patients received a LHRH agonist and/or a first‐generation androgen receptor antagonist, per our inclusion criteria. LHRH agonists included goserelin (146 patients, 95.4%) and buserelin (1, 0.7%). First‐generation androgen receptor antagonists included bicalutamide (136, 88.9%) and flutamide (4, 2.6%). Twelve patients (7.8%) underwent bilateral orchiectomy.

### 3.3 Overall survival

**FIGURE 1:**
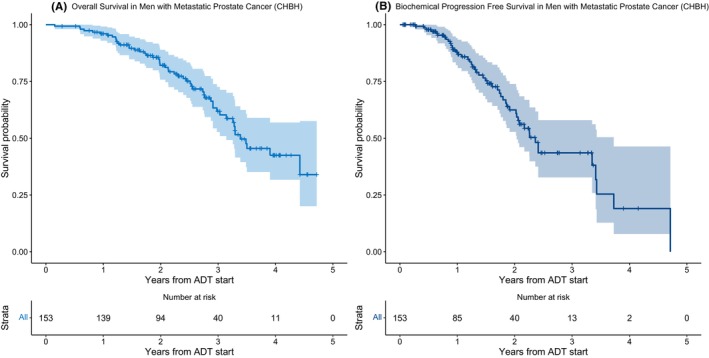
(A) Overall survival and (B) Biochemical progression‐free survival from date of ADT initiation in men with Stage IV prostate cancer enrolled in the MADCaP study at the Chris Hani Baragwanath Academic Hospital in Johannesburg, South Africa (2016–2020).

At the time of analysis, 52 (34%) of the 153 patients had died. Median OS from time of ADT initiation was 3.39 years (95% CI: 3.14%–noncalculable [NC]) (Figure 1A). Median follow‐up time for the OS analysis was 2.75 years. The estimated one‐, two‐, and three‐year OS from ADT start was 96.0% (95% CI: 92.9%–99.2%), 82.1% (95% CI: 75.8%–88.9%), and 61.9% (95% CI: 52.7%–72.6%), respectively.

Median OS from time of PCa diagnosis was 3.55 years in our cohort (95% CI: 3.38–NC). In the broader cohort of all 200 patients with Stage IV PCa, the median OS from time of PCa diagnosis was 3.38 years (95% CI: 3.01%–4.55%).

### 3.4 Biochemical progression‐free survival

The median bPFS was 2.36 years (95% CI: 2.03–3.73) (Figure 1B). The estimated one‐, two‐, and three‐year bPFS were 87.9% (95% CI: 82.%2–94.1%), 62.5% (95% CI: 52.8%–73.8%), and 43.6% (95% CI: 32.7%–57.9%), respectively.

### 3.5 Multivariate analyses

Our multivariate Cox model for OS included age at diagnosis, PSA at diagnosis, tumor volume, Gleason group, ALP and hemoglobin at diagnosis, PSA nadir (defined as ≤4 ng/mL and >4 ng/mL), and biochemical progression. We found that biochemical progression (HR 3.52, 95% CI: 1.85%–6.70%, *p*‐value < 0.001), nadir PSA level >4 ng/mL (HR 3.77, 95% CI 1.86%–7.62%, *p*‐value < 0.001), ALP >150 IU/dL at diagnosis (HR 3.09, 95% CI 164%–5.83%, *p*‐value < 0.001), and hemoglobin <13.5 g/dL at diagnosis (HR 2.90, 95% CI 1.28%–6.56%, *p*‐value = 0.01) were significantly associated with worse OS (Table 2). Age ≥ 65 years was also associated with worse OS, although the confidence intervals were wide (HR 2.40, 95% CI 1.04%–5.53%, *p*‐value 0.04).

**TABLE 2:** Cox proportional hazards ratio models of demographic and clinical factors associated with overall survival among men with stage IV prostate cancer who received ADT and were enrolled in the MADCaP study at the Chris Hani Baragwanath Academic Hospital in Johannesburg, South Africa (2016–2020).

Characteristic	Univariate HR	95% CI	*p*‐Value	Multivariate HR	95% CI	*p*‐Value
Age ≥65 years	1.41	0.68–2.91	0.4	2.40	1.04–5.53	0.04
PSA at diagnosis ≥ 100 ng/mL	2.16	1.24–3.77	0.006	0.87	0.45–1.69	0.7
Tumor volume ≥ 50%	1.90	1.03–3.52	0.041	1.29	0.65–2.54	0.5
Gleason group	1.47	1.07–2.01	0.016	1.21	0.82–1.79	0.3
ALP >150 IU/L	3.30	1.90–5.74	<0.001	3.09	1.64–5.83	<0.001
Hb <13.5 g/dL	4.01	1.94–8.29	<0.001	2.90	1.28–6.56	0.01
PSA nadir >4 ng/mL	4.35	2.28–8.29	<0.001	3.77	1.86–7.62	<0.001
PSA‐progression	4.69	2.64–8.35	<0.001	3.52	1.85–6.70	<0.001

Abbreviations: ALP = Alkaline Phosphatase; Hb = Hemoglobin; IPSS = International Prostate Symptom Score; PSA = Prostate‐Specific Antigen; UTI = Urinary Tract Infection.

## 4 DISCUSSION

The International Agency for Research on Cancer (IARC) predicts that yearly deaths from prostate cancer (PCa) will more than double in Sub‐Saharan Africa (SSA) from 47,000 in 2020 to more than 100,000 by 2040.^17^ Recognizing this large burden of disease, the National Cancer Control Network in the United States (NCCN) and Africa Cancer Coalition published the “Harmonized Guidelines for Sub‐Saharan Africa: Prostate Cancer Screening, Diagnosis, and Treatment” in 2019.^18^ These resource‐stratified guidelines recommend ADT plus a second‐generation anti‐androgen agent (apalutamide or abiraterone) or ADT plus docetaxel (for 6 cycles) as the preferred first‐line treatment in metastatic (Stage IV) castrate‐sensitive PCa, with ADT alone as an alternative (less‐preferred) recommendation. These recommendations are based on several recent phase III randomized controlled trials that have demonstrated improved survival for men with stage IV PCa with the addition of chemotherapy and/or second‐generation anti‐androgen agents to first‐line ADT.^19^, ^20^, ^21^, ^22^, ^23^, ^24^

Nonetheless, real‐world implementation of such cancer treatment guidelines is often very delayed or not possible in low‐resource settings. In this study, we report detailed real‐world demographic, clinical, and treatment data, as well as progression‐free and overall survival for a cohort of 153 Black African men who were diagnosed with Stage IV PCa at Chris Hani Baragwanath Academic Hospital (CHBAH) in Soweto, Johannesburg (South Africa) between March 22nd, 2016 and October 30th, 2020 and treated with ADT. In our cohort, the median OS from ADT initiation was 3.39 years. High ALP and anemia at diagnosis, PSA nadir >4 ng/mL after ADT, and biochemical progression were associated with shorter OS.

CHBAH is a state‐sector hospital with an academic affiliation and the largest public hospital in SSA. The catchment area of CHBAH is the poor and mostly Black population of Soweto township, which is reflected in the low median monthly income of the described cohort (<1850 ZAR per month, equivalent to <$132). Due to resource constraints, first‐line treatment for patients with Stage IV PCa at CHBAH is restricted to conventional ADT (orchiectomy, LHRH agonists, and first‐generation androgen receptor antagonists). During the study period, patients with Stage IV PCa who progressed on ADT were referred to Charlotte Maxeke Johannesburg Academic Hospital (CMJAH) for chemotherapy (docetaxel) in the second‐line setting. CMJAH is a sister facility 20 km away from Soweto, and many patients cannot afford the taxi far to get to apptointments at CMJAH; this explains the likely very low rate of patients in our cohort who received second‐line docetaxel (data not shown).

The Soweto Comprehensive Cancer Center at CHBAH opened in 2021 (sponsored by a private pharmaceutical company), and second‐line docetaxel is now more readily available to patients treated at CHBAH. However, chemotherapy in the first‐line setting for stage IV PCa patients remains rare, and second‐generation anti‐androgens (abiraterone, enzalutamide, and apalutamide) are still not available due to their high costs. There are significant efforts within the Departments of Urology and Oncology at CHBAH to make these treatments available to a limited number of selected, high‐risk patients, but as of the writing this paper, no such budget has been approved.

Comparison of the South African cohort described in this study to other published studies from SSA demonstrates both similarities and unique characteristics. In 2021, Seraphin et al.^25^ published a population‐based registry study of presentation, patterns of care, and outcomes of patients with PCa in SSA. The study included 693 patients with PCa from 10 countries in SSA (Cote d'ivoire, Ethiopia, Mali, Congo, Zimbabwe, Benin, Kenya, Uganda, Mozambique, and Namibia). However, detailed tumor characteristics and treatment information were available for only 365 patients (“traced cohort”) and survival information for 491 patients. Like SA, none of these 10 countries have organized or opportunistic PCa screening programs, and all men included in the study were symptomatic. Among the 365 patients in the traced cohort, 37.3% (*n* = 136) were metastatic, which is a higher percentage than in our cohort, where we found that 22.8% of men with complete staging and 18.2% of all men diagnosed with PCa had metastatic or stage IV disease at diagnosis. We hypothesize that this difference reflects the higher socioeconomic status of SA compared to the countries included in the Seraphin et al. cohort and the robust South African healthcare system, where men may present to care earlier or may be evaluated more expeditiously for symptoms compared to other countries in SSA. Alternatively, men in East, West, and Central Africa may have more aggressive PCa disease compared to men in Southern Africa, although little data exists to support such biological differences among African men, and the Seraphin et al. cohort does include men from Zimbabwe, which is in Southern Africa. Follow‐up studies are warranted to test these hypotheses.

The median age of our cohort is comparable to that in the Seraphin et al.^26^ publication (68 years, IQR 64–73 and 69 years, IQR 63–75, respectively) and older compared to the 2017–2019 SEER cohort from the United States (median age 62 IQR, 56–69 years for Black men and 65, IQR, 59–71 years White men in the United States). This age discrepancy likely reflects prostate cancer screening patterns in the United States and lack of screening in SSA. Comparisons of T‐stage, PSA, and highest Gleason score at presentation between our cohort and the Seraphin et al.^25^ publication are difficult because of the high degree of missing data for these variables, even in the traced cohort (55.9% no T‐stage, 59.6% no PSA at diagnosis, 56.5% no Gleason score). The relatively low degree of missing data in our cohort makes it unique compared to other published cancer data from SSA.

With regards to treatment, of the 136 patients with metastatic PCa in the Seraphin et al. cohort, 22.8% (*n* = 31) did not receive any cancer‐directed therapy, 59.6% (*n* = 81) received ADT, with or without other cancer‐directed therapy, and 17.7% (*n* = 24) received other cancer‐directed therapy without ADT (chemotherapy, surgery, or external beam radiation). In comparison, only 8 out of 200 men with metastatic PCa (4%) did not initiate ADT in our cohort, while an additional 39 men (19.5%) initiated ADT but were lost to follow‐up before at least one PSA level was drawn ≥12 weeks after ADT start. This data demonstrates a higher rate of treatment and/or treatment uptake with ADT among South African men than what is reported from cancer registries in 10 countries across SSA. Another striking difference between the Seraphin et al. cohort and our study is a significantly higher rate of surgical ADT in other SSA countries – 45.6% of patients with metastatic PCa who received ADT underwent surgical orchiectomy, compared to just 7.2% in the South African cohort presented here. A similarity between the previously published cohort and our study is the very low rate of treatment with chemotherapy among men with metastatic PCa; none of the men in our study were treated with chemotherapy in the first‐line setting, and in the Seraphin et al. study, although 77.2% (*n* = 105) of men with metastatic PCa received cancer‐directed therapy (with or without ADT), only 1 patient received chemotherapy.

Seraphin et al. present 1‐, 3‐, and 5‐year overall survival data for the metastatic subgroup (*n* = 136) of 61.2% (95% CI: 52.2%–70.2%), 25.8% (95% CI: 16.4%–35.2%), and 14.7% (95% CI: 5.0%–24.5%), respectively. Among 99 patients with metastatic PCa and ≥12 weeks of follow‐up in the Seraphin et al. cohort, 2‐year OS was approximately 50% among patients who received cancer‐directed therapy, compared to 20% among those who did not. Non‐receipt of cancer‐directed therapy was associated with worse survival in a multivariable analysis by Seraphin et al. In comparison, in our cohort of 153 men treated with ADT in Soweto, Johannesburg, 2‐year OS from ADT start was 82.1% (95% CI: 75.8%–88.9%).

In our cohort of men with metastatic PCa who received ADT, we had relatively low rates of missing data and sufficient patients with adequate follow‐up to determine OS, biochemical progression‐free survival, and predictors of death. Identifying patients with Stage IV PCa at high risk of death is important in resource‐constrained healthcare settings, as it allows clinicians to select which patients require more aggressive treatment. With annual costs in the United States of approximately $35,000–$50,000 for second‐generation anti‐androgen agents, these drugs are unaffordable in most low‐resource settings.^27^ On the other hand, generic docetaxel in SA costs approximately $10–20 per dose, making it a realistic treatment option for patients with unfavorable risk Stage IV PCa and good functional status and supporting the inclusion of this treatment strategy as a preferred option in the resource‐stratified NCCN guidelines.^28^ Our study, as well as a population‐based cancer registry study of patients with PCa in 10 other countries in SSA, demonstrate that chemotherapy for stage IV PCa is severely underused in this setting.^25^ In contrast, a retrospective cohort study of 874 men diagnosed with PCa at the Uganda Cancer Institute was published in 2020 and showed that 43.94% of men were treated with chemotherapy (mix of men with localized and metastatic PCa).^29^ The most commonly used chemotherapy agents were cabazitaxel and docetaxel, in keeping with NCCN recommendations.^18^, ^29^ Based on our study, there is clearly room within the South African public health system to increase compliance with the resource‐stratified NCCN guidelines for stage IV PCa.

The findings of our study provide critical data on the efficacy of ADT as first‐line treatment for patients with Stage IV PCa in SA and provide a framework to help clinicians select which patients may benefit most from the addition of upfront chemotherapy to ADT. In particular, ALP >150 IU/L or Hb <13.5 g/dL at diagnosis and PSA nadir >4 ng/mL 12 weeks or more after ADT start were all significantly associated with shorter mOS among men treated with ADT alone. High ALP and low Hb are considered markers of mPCa disease severity and have been associated with worse OS in non‐African cohorts as well.^30^, ^31^ High PSA nadir is similarly associated with poor response to treatment and has been shown to correlate with poor prognosis in other studies.^32^

The bPFS in our cohort was 2.36 years (95% CI: 2.03%–3.73%). Our multivariate model for mOS demonstrated that having a biochemical progression event was significantly associated with OS (HR 3.52, 95% CI: 1.85–6.70). In Stage IV PCa patients who do not meet high‐risk criteria for upfront chemotherapy, biochemical progression should prompt addition of chemotherapy to ADT.

To our knowledge, this is the first large study to evaluate the factors associated with OS in Black African men with Stage IV PCa treated with ADT. Bello et al published a small study evaluating factors associated with OS among 48 Nigerian men with metastatic castrate resistant PCa.^33^ In a study including both patients with localized and metastatic PCa from Uganda (*n* = 36 patients with stage IV disease), Yahaya et al.^34^ found that Gleason score and LVI were independent predictors of survival.

### 4.1 Strengths and limitations

Study strengths include a large cohort with extensive demographic, treatment, laboratory, and survival information on all patients. Serial PSA levels are often not available from PCa cohorts from SSA; thus, our dataset is unique in that it enabled us to accurately estimate bPFS. Our analysis provides important information regarding OS and bPFS among men with mPCa.

Study limitations include significant censoring of patients, especially in the bPFS analysis, where censoring occurred at date of last recorded PSA. This resulted in early censoring of patients lost to follow‐up after only 1 or 2 visits, before progression is expected. Additionally, PSA levels were not drawn at standardized time intervals, and follow‐up times varied between 6 and 12 months, which could have biased results. Finally, we excluded patients with incompletely staged PCa, many of whom likely had Stage IV PCa, which could have further biased our results.

## 5 CONCLUSIONS

Stage IV PCa in SA is treated with ADT alone, without chemotherapy or second‐generation anti‐androgen agents. Survival outcomes in our cohort are similar to those of patients with Stage IV PCa in higher‐resource countries treated with ADT alone and in ADT‐alone groups of clinical trials. High ALP, anemia at diagnosis, PSA nadir >4 ng/mL, and biochemical progression are associated with shorter OS in Black South Africans with Stage IV PCa treated with ADT. These factors should be used to identify patients for treatment escalation, including the early use of chemotherapy.

## AUTHOR CONTRIBUTIONS

**Yoanna S. Pumpalova:** Conceptualization (lead); data curation (lead); formal analysis (lead); writing – original draft (lead); writing – review and editing (lead). **Adarsh Ramakrishnan:** Data curation (supporting); formal analysis (supporting); writing – original draft (supporting); writing – review and editing (supporting). **Michael May:** Formal analysis (supporting); writing – original draft (supporting); writing – review and editing (supporting). **Audrey Pentz:** Data curation (supporting); investigation (supporting); project administration (supporting). **Shauli Minkowitz:** Conceptualization (supporting); investigation (supporting); writing – review and editing (supporting). **Sean Doherty:** Investigation (supporting); writing – review and editing (supporting). **Elvira Singh:** Data curation (supporting); formal analysis (supporting); methodology (supporting). **Wenlong Carl Chen:** Data curation (supporting); project administration (supporting); writing – review and editing (supporting). **Timothy R. Rebbeck:** Conceptualization (supporting); funding acquisition (lead); supervision (supporting); writing – review and editing (supporting). **Alfred I. Neugut:** Methodology (supporting); supervision (supporting); writing – review and editing (supporting). **Maureen Joffe:** Funding acquisition (supporting); investigation (supporting); project administration (lead); writing – review and editing (supporting).

## FUNDING INFORMATION

This study was funded by an NIH grant (U01‐CA184374) awarded to Dr. Rebbeck and MADCaP co‐investigators. The study was also supported by the National Institutes of Health, National Cancer Institute CCSG P30‐CA13696 and R38 CA231577. The content is solely the responsibility of the authors and does not necessarily represent the official views of the National Institutes of Health. Dr. Pumpalova was also partially funded through an ASCO/Conquer Cancer Foundation Young Investigator Award (2021‐2022).

## CONFLICTS OF INTEREST STATEMENT

Alfred Neugut: Otsuka, United Biosource Corp, Hospira, Eisai, and GlaxoSmithKline (Consulting/advisory relationship); EHE Intl (Scientific Advisory Board); Otsuka (Research Funding); Yoanna Pumpalova: Pfizer (Ownership Interest). The other authors indicated no financial relationships.

## ETHICS STATEMENT

Approval was obtained from the Human Research Ethics Committee (Medical) at the University of the Witwatersrand (certificate numbers M150934 and M220673) and Harvard University. The procedures used in this study adhere to the tenets of the Declaration of Helsinki.

## CONSENT TO PARTICIPATE

Informed consent was obtained from all individual participants included in the study.

## PRIOR PRESENTATIONS

This work was presented as a poster at the American Society for Clinical Oncology Annual Conference in Chicago, IL, USA in June 2022.

## Data Availability

The datasets generated during an/or analyzed during the current study are available from the corresponding author in reasonable request.
